# Correlation with a limited set of behavioral niches explains the convergence of somatic morphology in mygalomorph spiders

**DOI:** 10.1002/ece3.9706

**Published:** 2023-01-09

**Authors:** Jeremy D. Wilson, Jason E. Bond, Mark S. Harvey, Martín J. Ramírez, Michael G. Rix

**Affiliations:** ^1^ Biodiversity and Geosciences Program Queensland Museum Collections and Research Centre Hendra Queensland Australia; ^2^ Department of Entomology and Nematology University of California Davis California USA; ^3^ Collections and Research Western Australian Museum Welshpool Western Australia Australia; ^4^ School of Biological Sciences University of Western Australia Crawley Western Australia Australia; ^5^ Museo Argentino de Ciencias Naturales Consejo Nacional de Investigaciones Científicas y Técnicas Buenos Aires Argentina

**Keywords:** adaptive landscape, chronogram, comparative phylogenetic methods, morphological convergence, niche dynamics, phylogram, supertree

## Abstract

Understanding the drivers of morphological convergence requires investigation into its relationship with behavior and niche space, and such investigations in turn provide insights into evolutionary dynamics, functional morphology, and life history. Mygalomorph spiders (trapdoor spiders and their kin) have long been associated with high levels of morphological homoplasy, and many convergent features can be intuitively associated with different behavioral niches. Using genus‐level phylogenies based on recent genomic studies and a newly assembled matrix of discrete behavioral and somatic morphological characters, we reconstruct the evolution of burrowing behavior in the Mygalomorphae, compare the influence of behavior and evolutionary history on somatic morphology, and test hypotheses of correlated evolution between specific morphological features and behavior. Our results reveal the simplicity of the mygalomorph adaptive landscape, with opportunistic, web‐building taxa at one end, and burrowing/nesting taxa with structurally modified burrow entrances (e.g., a trapdoor) at the other. Shifts in behavioral niche, in both directions, are common across the evolutionary history of the Mygalomorphae, and several major clades include taxa inhabiting both behavioral extremes. Somatic morphology is heavily influenced by behavior, with taxa inhabiting the same behavioral niche often more similar morphologically than more closely related but behaviorally divergent taxa, and we were able to identify a suite of 11 somatic features that show significant correlation with particular behaviors. We discuss these findings in light of the function of particular morphological features, niche dynamics within the Mygalomorphae, and constraints on the mygalomorph adaptive landscape relative to other spiders.

## Graphical abstract

We reconstruct the evolution of burrowing behavior in mygalomorph spiders, compare the influence of behavior and evolutionary history on their somatic morphology, and test hypotheses of correlated evolution between specific morphological features and behavior. We find that shifts across the simple adaptive landscape of the Mygalomorphae are common in their evolutionary history and that their somatic morphology is heavily influenced by behavior, with taxa inhabiting the same behavioral niche often more similar morphologically than more closely related but behaviorally divergent taxa. We identify a suite of 11 somatic features that show significant correlation with particular behaviors.

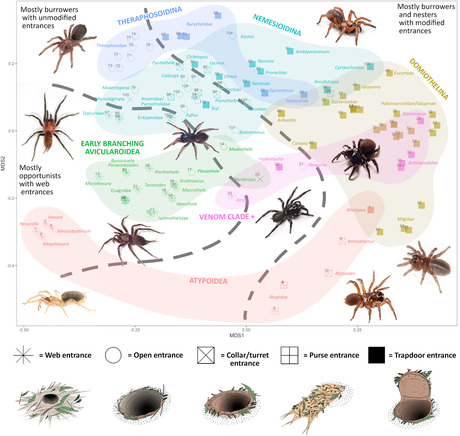

## 1 INTRODUCTION

Convergent morphological evolution, the independent evolution of similar phenotypes, has long fascinated biologists because it represents natural replicates of the evolutionary process (Darwin, 1859; McGhee, 2011). It is traditionally seen as straightforward evidence of adaptation to similar environmental pressures (Mayr, 2013; Simpson, 1953), yet recent studies have highlighted other contributing factors and encouraged a more nuanced view (Conway Morris, 2010; Losos, 2011; Stayton, 2015). Firstly, the important role of evolutionary constraints has been emphasized (Conway Morris, 2010; McGhee, 2011). Without constraints on the adaptive landscape of an organism, the same niches need never arise, and even when they do, lineages may evolve different traits to overcome the same niche‐specific function (Losos, 2011). Furthermore, when constraints are strong enough, morphological convergence may occur for reasons other than adaptation to environmental pressures, or simply by chance (Losos, 2011; Stayton, 2008). Understanding the drivers of morphological convergence in a group, therefore, requires not only identification of the phenomenon itself but also further investigation into its relationship with behavior and niche space. Such broad, combined analyses of morphology and behavior in turn provide insights into the evolutionary dynamics, functional morphology, and life history of the study group, as demonstrated in recent analyses on birds (Pigot et al., 2020), mammals (Sansalone et al., 2020), and marine tetrapods (Kelley & Motani, 2015), yet equivalent studies on invertebrates are few (Ceccarelli et al., 2019).

The spider infraorder Mygalomorphae currently contains 31 families of relatively large, robust spiders that generally live sedentary lives in permanent retreats or burrows (Bond et al., 2012; Opatova et al., 2020; Raven, 1985). It includes species commonly known as tarantulas, trapdoor spiders, and funnel‐web spiders. The group has a tumultuous taxonomic history, but the first major work, bringing some order to the chaos, was that of Raven (1985). This exhaustive morphological review, and the accompanying cladistics‐based phylogeny, served as the foundation of mygalomorph systematics for two decades and remains the most complete synopsis of mygalomorph morphology available. However, the implementation of molecular methods has revealed phylogenetic relationships in stark discordance with those deduced from morphology: over half the traditional families were revealed to be paraphyletic and accepted interfamilial relationships have changed dramatically (Bond et al., 2012; Hedin et al., 2018, 2019; Hedin & Bond, 2006; Montes de Oca et al., 2022; Opatova et al., 2020). With the recent taxon‐rich, genomic phylogeny of Opatova et al. (2020), accepted mygalomorph relationships have largely stabilized. What is still required, however, is a reconciliation of mygalomorph morphology and behavior with this new phylogeny, to understand the broad evolutionary patterns in the group that were previously obscured by taxonomic and phylogenetic uncertainty and instability.

One pattern that is often proposed to explain the discordance between morphological and molecular hypotheses of mygalomorph relationships is convergence in somatic morphology associated with life history characteristics (Hedin et al., 2019; Hedin & Bond, 2006; Opatova et al., 2020). The retreats of these spiders come in a diversity of forms including, among many others: funnel‐like silken retreats built in crevices with extensive capture webs; burrows in the ground with or without a trapdoor entrance; and short nests constructed against tree trunks (Coyle, 1986). Reconstructions of these “behavioral niches” on new molecular phylogenies have consistently found that each has evolved several times across mygalomorphs (Hedin et al., 2019; Opatova et al., 2020). Intuitive associations between particular niches and somatic characters have long been recognized, for example, between elongate posterior lateral spinnerets and the construction of capture webs (Chamberlin & Ivie, 1945; Eskov & Zonshtein, 1990) and between strong lateral “digging spines” on the anterior legs and the construction of burrows (Goloboff, 1993; Raven, 1985). However, to date, neither the overarching influence of convergence on mygalomorph morphology nor specific patterns of correlation with behavior of any morphological feature have ever been specifically tested.

The aim of this study is to characterize what is potentially a major evolutionary trend in the Mygalomorphae – the convergence of somatic morphology in correlation with the behavioral niches inhabited by the group. Using a selection of recent, robust genomic phylogenies available in the literature, we construct a genus‐level phylogram and chronogram, and a taxon‐rich supertree. Next, we score all genera in these trees for a discrete dataset of 2 behavioral and 55 somatic‐morphological characters. We then perform the most detailed reconstruction of behavioral niche in the Mygalomorphae to date, to understand patterns of convergence in behavioral niche and the association between retreat type and retreat‐entrance type. Next, to compare the influence of behavioral niche and evolutionary history on general somatic morphology, we perform non‐metric multidimensional scaling on the full morphological dataset, visualizing somatic variation in morpho‐space. Finally, we test for correlation between a subset of morphological features and particular behavioral niches to shed light on the function of these features and the drivers of convergence in the Mygalomorphae.

## 2 MATERIAL AND METHODS

### 2.1 Phylogeny selection and supertree construction

We constructed three genus‐level phylogenies using publicly available data. For analyses requiring informative branch lengths, we used the RAxML (Stamatakis, 2014) phylogram and treePL (Smith & O'Meara, 2012) chronogram of Opatova et al. (2020) both generated using an anchored hybrid enrichment (AHE) dataset consisting of 472 loci. We used the R‐package *ape* (Paradis & Schliep, 2019) to prune these trees down to a single representative per genus and a single outgroup (*Liphistius*: Liphistiidae), resulting in an 89‐taxon genus‐level chronogram and phylogram.

**FIGURE 1:**
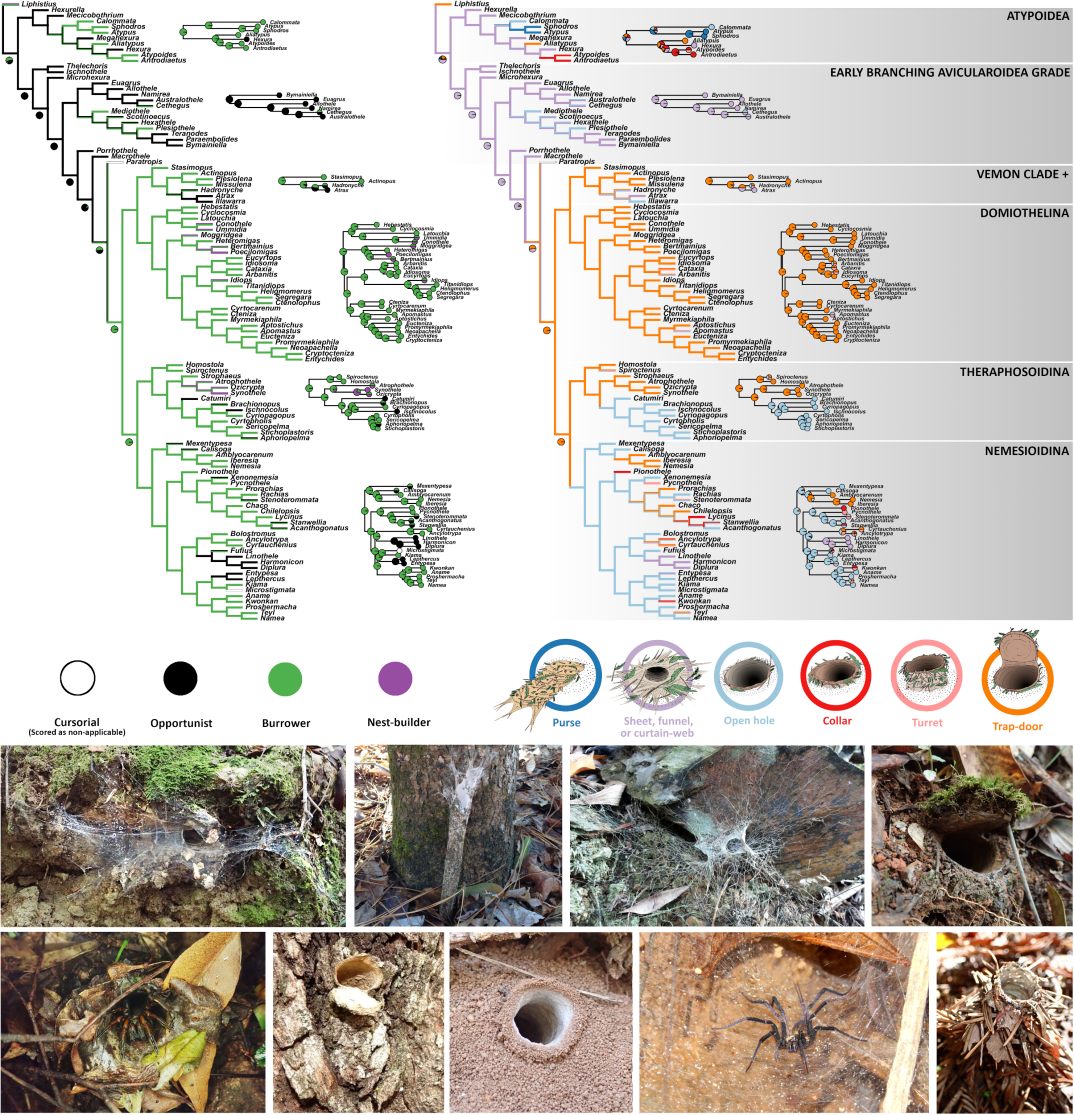
Evolution of behavioral niche in the Mygalomorphae. The top panels show ancestral state reconstructions of retreat construction method (left) and retreat‐entrance type (right) with the key to states below the reconstructions. Complete cladograms show reconstructions using maximum parsimony (MP) on our supertree, and partial phylograms and pie charts show relevant sections of the maximum‐likelihood (ML) reconstructions, conducted on the genus‐level phylogeny. The bottom panel shows examples of different behavioral niches, with the genus, niche, and photographer as follows (clockwise from top left): *Namirea* (Euagridae), opportunist + web entrance, J. Wilson; *Sphodros* (Atypidae), burrower + purse‐web entrance, R. Deans; *Hadronyche* (Atracidae), opportunist + web entrance, M. Rix; *Euoplos* (Idiopidae), burrower + trapdoor entrance, J. Wilson; *Atypoides* (Antrodiaetidae), burrower + turret entrance, C. Raspet; *Linothele* (Dipluridae), opportunist + web entrance, K. Venegas Valancia; *Kwonkan* (Anamidae), burrower + collar entrance, T. Barbin; *Migas* (Migidae), nest‐builder + trapdoor entrance, G. Walter; and *Arbanitis* (Idiopidae), burrower + open entrance, J. Wilson.

For analyses not requiring informative branch lengths, we constructed a more inclusive supertree using several recent mygalomorph‐focused genomic phylogenies. We downloaded the maximum‐likelihood phylogenies of Opatova et al. (2020) – Mygalomorphae‐focused (AHE data); Hedin et al. (2018) – Atracidae‐, Actinopodidae‐, and Hexathelidae‐focused (ultra‐conserved elements [UCE]); Hedin et al. (2019) – Atypoidea‐focused (UCE); and Montes de Oca et al. (2022) – Nemesoidina‐focused (AHE). For the latter, the raw tree file was not available, so we generated a new maximum‐likelihood phylogeny using IQtree (Nguyen et al., 2015) using the alignment and partition files from the study (Appendix S1). We pruned these phylogenies down to a single representative per genus, rooted them, and used them as input trees for supertree construction using matrix representation with parsimony (MRP) in the R‐package *phangorn* (Schliep, 2011), resulting in a 110‐taxon final supertree (Figure 1). The supertree topology was uncontroversial except in the position of the Venom Clade + Stasimopidae (from here on referred to as the Venom Clade+), which was recovered as either sister to the Domiothelina or of the clade including the Domiothelina and Crassitarsae. We chose to use the first of these topologies as it agrees with Opatova et al. (2020), which represents the most robust mygalomorph phylogeny currently available.

### 2.2 Behavioral and morphological character scoring

By combining a semi‐exhaustive literature review with exemplar cross‐checking, we then scored 2 behavioral characters and 55 morphological characters (see Appendix S2 for character information, and see Wilson, Bond, et al., 2022, for character matrix, relevant literature, and exemplar specimen information) for all 110 genera in the supertree. Behavioral characteristics relate to retreat construction method and retreat‐entrance type and are defined below. To score these characters, we made extensive use of Coyle (1986), which remains the most thorough review of mygalomorph burrowing behavior to date, and then cross‐checked this with taxon‐specific literature – see Wilson, Bond, et al. (2022) for a complete list of the literature reviewed while scoring taxa. The 55 morphological characters are all somatic, macro‐morphological features (Appendix S2). These were scored exclusively from adult females because adult male morphology is at least partially adapted for the terrestrial dispersal phase that they undergo, whereas female morphology is more representative of the general morphology of the species (in that juveniles of both sexes resemble adult females) and is presumably adapted to the sedentary lifestyle of the species. Most of our morphological characters correspond closely with those scored in previous morphological analyses of the Mygalomorphae (Bond et al., 2012; Bond & Opell, 2002; Goloboff, 1993, 1995; Raven, 1985), but we have restructured characters following the logic for character/state structure outlined by Sereno (2007) and modified character and state definitions to decrease ambiguity. These previous studies were used extensively during character scoring, with taxon‐specific literature and exemplar specimens then cross‐checked when available (Wilson, Bond, et al., 2022). Many mygalomorph genera are polymorphic for the behaviors and morphological characters scored here and were scored as such in the dataset (Wilson, Bond, et al., 2022). Likewise, for some poorly known genera, not all characters could be scored from the literature and exemplars, and some data are therefore missing for these taxa.

#### 2.2.1 Behavioral characters

1. *Retreat construction method: Opportunist* – taxa that usually inhabit existing spaces (e.g., cracks and overhangs in embankments, spaces under rocks and within logs) rather than digging/constructing a retreat = 0; *obligate burrower* – taxa that usually dig their own tubular burrow directly into the substrate = 1; *nest‐builder* – taxa that construct short, silken nests, which are attached directly to the substrate (often on trees, cave walls, or sometimes directly to the ground) = 2.
2. *Retreat entrance, type: web* – extensive use of silk outside the entrance to the retreat to form a flat sheet, a funnel, or a space/curtain web = 0*; open* – an unmodified, circular opening to the retreat (which may temporarily be covered with silk or soil by the spider) = 1*; turret* – an entrance that is open, but modified to extend from the substrate through the use of silk and/or soil = 2; *collar* – an entrance that is closable through the use of a silken collar that collapses inward = 3*; trapdoor* – an entrance that is closed with a “door” constituting an asymmetrical extension of the burrow lining (often mixed with soil and/or humus fragments), allowing the demarcation of one side of the burrow as the “hinge” side = 4; and *purse* – an extension of the burrow lining that lies along the substrate or is attached vertically to a surface, is rough and camouflaged, through which the spider ambushes prey = 5.

### 2.3 Analyses

To understand the evolution of behavioral niche in the Mygalomorphae and identify cases of niche convergence, we conducted ancestral state reconstructions (ASR) on our two behavioral characters. We compared the results of two methods: we conducted a maximum‐likelihood (ML) approach (Pagel, 1999) on the genus‐level phylogram and chronogram using the *corHMM* R package (Beaulieu et al., 2021), and the maximum‐parsimony (MP) approach (Swofford & Maddison, 1987) on the supertree using Mesquite v3.51 (Maddison, 2008). For the ML reconstructions, we compared AICc scores across both alternate branch length sets (i.e., the chronogram and phylogram, see Wilson, Mongiardino Koch, et al., 2022) and across alternate state‐transition models in which all transition rates were equal (equal rates – ER), transition rates were estimated separately for each pair of states, but were equal in both directions for each (symmetrical – SYM), and transition rates were allowed to vary between all state pairs and directions (all rates different – ARD). We then chose the branch length set and model that minimized AICc (Appendix S3). Currently, using a phylogram for ancestral state reconstruction rather than a chronogram remains controversial. However, studies have now shown that rates of morphological change can also strongly correlate with rates of molecular change (Seligmann, 2010), suggesting that a phylogram may be more appropriate for ASR of morphological characters in some cases.

Next, to visualize how mygalomorph somatic morphology relates to the behavioral niches that they inhabit, we conducted non‐metric multidimensional scaling (NMDS) using the complete 55‐character morphological dataset, revealing the position in two‐dimensional “morpho‐space” of all genera included in the study and its relationship with behavior. This analysis involved first calculating the Gower similarity coefficient (Gower, 1971) between all pairs of taxa based on the morphological characters, using the *Claddis* R‐package (Lloyd, 2016) before using the resultant pairwise similarity matrix to conduct the NMDS analysis, using the R‐package *vegan* (Oksanen et al., 2013).

Finally, to identify the specific morphological features associated with different behavioral niches, and thereby better understand their function, we conducted a series of phylogenetic tests for correlated evolution between morphological features and behavior (Table 1). A morphological feature was tested for correlation with behavior if: (i) an association between the feature and behavior has been proposed previously in the literature; (ii) the function of the feature is known and is tied with a particular behavior; or (iii) a strong association between a feature and behavior was perceived while scoring characters for this study. We tested all selected morphological features for correlation with five key behaviors, all of which have evolved multiple times in mygalomorphs: (a) construction of a web (sheet, funnel, or curtain) at the entrance to the retreat; (b) opportunistic retreat construction (as opposed to construction of a burrow or nest); (c) construction of a burrow; (d) structural modification of the retreat entrance (with a purse, collar, turret, or trapdoor); and (e) construction of a hinged trapdoor at the retreat entrance.

**TABLE 1:** Morphological features tested for correlation with behavior, with a justification for their inclusion.

Features	Justification
Spinnerets: Elongate posterior lateral spinnerets (C11) Widely separated spinnerets (C2) Pseudo‐segmented apical segment of posterior lateral spinnerets (C10) Short apical segment of posterior lateral spinnerets (C9)	An association between “Dipluridae type” posterior lateral spinnerets, which are elongate and widely separated, and the construction of webs (sheet, funnel, or curtain) has been proposed previously (Chamberlin & Ivie, 1945; Coyle, 1971; Eskov & Zonshtein, 1990). In some taxa with this spinneret type (and none without it), the spinnerets are pseudo‐segmented, so this is also presumably associated with the same behavioral niche. At the opposite end of the spectrum, spinnerets with very short apical segments (traditionally called “domed” or “triangular” apical segments) show a clear pattern of association with burrowing spiders, many of which modify their burrow entrance
Chelicerae and mouthparts: Presence of a rastellum (C51) Presence of a serrula (C43)	Observations of burrowing behavior indicate that the rastellum is used during burrow excavation and/or for modifying the burrow entrance (Coyle, 1971, 1981; Nascimento et al., 2021). Although the function of the serrula in Mygalomorphae is not well established, we observed a potential association with spiders that construct opportunistic retreats and/or that do not construct a burrow. This is perhaps most evident in the Atypoidea, where the serrula is present in all species that show opportunistic retreat‐construction habits (*Hexurella, Mecicobothrium, Megahexura*, and *Hexura*), and is absent in all genera that burrow (all Atypidae*, Aliatypus, Atypoides*, and *Antrodiaetus*)
Chaetotaxy of the anterior legs: Digging spines on legs I–II (C18) Presence of scopulae on the anterior tarsi/metatarsi (C20)	Strong lateral spines on at least metatarsi I–II, but usually also the tarsi and tibiae (previously called “digging spines”) have previously been associated with burrowing and/or trapdoor construction, and potentially prey capture (Raven, 1985). However, we observed that even in burrowing spiders, species with scopulae rarely possess these spines. We therefore hypothesized a positive correlation between digging spines and burrowing behaviors, but only when scopulae were not present. Scopulae have been studied extensively (Pérez‐Miles et al., 2017; Wolff et al., 2013; Wolff & Gorb, 2012), with their major functions proposed as prey capture and locomotion. Pérez‐Miles et al. (2017) identified an association between scopulae and particular burrowing behaviors, so we also tested this feature for correlation here as well. Characters of the tarsal extremities were not analyzed, as most showed no obvious association with behavioral niche (e.g., claw tufts and biserially dentate paired claws appear to have few or single origins and have rarely been lost despite the groups in which they are found inhabiting a range of behavioral niches) and we believe more subtle characters of claw dentition deserve more detailed attention prior to tests of association with behavior
Chaetotaxy of the posterior legs: Leg III being thicker and at least as long as leg II (C13) Spines of leg III mostly dorsal (C14) Patella III with pro‐dorsal patch of >3 thorn‐like setae (C15)	Behavioral observations have shown that in burrowing spiders, leg III, and the posterior legs more generally, are used to anchor the spider in place in the burrow and for propulsion (presumably during prey capture; Bond & Coyle, 1995; Coyle, 1981; Decae & Bosmans, 2014). We have observed that in burrowing spiders the posterior legs are generally larger relative to the anterior legs, have spines positioned mostly dorsally, and may be modified in other ways, either possessing a tibial saddle (a concave, asetose section of cuticle) or a patch of thorn‐like spines on pro‐dorsal patella III (and sometimes also on patella IV). We hypothesized that these characters are probably correlated with burrowing or entrance modification of some kind, and tested all of them except the tibial saddle because this character is rare and restricted to relatively closely related taxa
Eye group: Presence of a common tubercle (C25) A compact, rectangular eye group (C22–23) A wide‐eye group (C22) Anterior lateral eyes in an advanced position relative to anterior median eyes (C23)	If we consider the “standard” eye group to be a compact rectangle on a common tubercle, then this is modified in several ways within the Mygalomorphae. Firstly, the tubercle may be absent. Secondly, the formation of the eyes may be modified, with two common modifications being a widening of the eye group (e.g., in Actinopodidae and Migidae) or the anterior lateral eyes being positioned far advanced of the others (e.g., in Barychelidae and some Idiopidae). We observed that all modifications mentioned above are more common in spiders that modify the burrow entrance, and virtually never occur in non‐burrowers, and therefore tested these characters for correlation with behavior

*Note*: C‐numbers listed after each feature denote the relevant character in the morphological character matrix (Appendix S2). See Figure 3 for representations of these features on spider schematic representations.

We tested the hypotheses in two steps. Firstly, we used the pairwise comparisons method (Maddison, 2000; Read & Nee, 1995) to test correlation between each morphological feature and all five behaviors. This method was applied as a stringent first pass because it is relatively robust to the “pseudoreplication problem” that causes many other phylogenetic correlation tests to identify significant correlation in questionable scenarios (see Maddison & FitzJohn, 2015). Because this method does not consider branch lengths, it was conducted using the supertree to benefit from the additional taxa. The analysis was performed twice for each character, the first time using only pairs that contrasted in both characters (i.e., morphology and behavior), and the second time using pairs that varied in at least one of the two characters (i.e., morphology and/or behavior; Maddison, 2000; Read & Nee, 1995). For each approach, we identified 1000 alternative pairing schemes, and from these, we took the highest possible *p*‐Value as our significance threshold, thereby reducing the chance of type‐1 error.

After using this first step to identify significant cases of correlation, we then analyzed these cases using maximum‐likelihood methods (sensu Pagel, 1994). We again used *corHMM* to estimate the likelihood and AICc values of a total of 22 different Markov models for each morphology/behavior character pair. These included models of independence (i.e., no correlation), morphological dependence on behavior, behavioral dependence on morphology, and morphological/behavioral interdependence (i.e., three different models of correlation), resulting in four different categories of dependence. For each of these four categories, we included models constraining transition rates for none, one, or both characters to be equal in both directions between states, leading to four models per dependence category: ER–ER, ER–ARD, ARD–ER, ARD–ARD, and a total of 16 “standard” models in our set. Boyko and Beaulieu (2022) recently demonstrated that the use of hidden Markov models (HMM), which help account for rate heterogeneity in the characters in question, can reduce the risk of false positives in maximum‐likelihood tests of correlation. As such, for all four independent models in our “standard” set, we included a counterpart with hidden rate categories (two rate categories for each state), leading to a total of 20 models tested per character combination. We then identified the best‐fitting model for each category of dependence using AICc, and compared the fit of these four models using delta‐AICc, to assess their relative strength (see Table 2). A lower delta‐AICc value indicates a better model fit relative to the best model, with the best model scoring a delta‐AICc of 0.

## 3 RESULTS

### 3.1 Reconstruction of behavioral niche

Ancestral state reconstructions of retreat type and entrance type resulted in largely consistent and complementary evolutionary patterns (Figure 1), and there are clear associations between the two: web‐building taxa are almost all opportunists, taxa that modify their burrow entrance with a purse, turret, collar, or trapdoor are almost always burrowers or nest‐builders, and nest‐builders always have a trapdoor.

In the MP analyses, the ancestral mygalomorph and the ancestors of both the Atypoidea and the Avicularioidea were recovered as opportunists with web entrances (funnel, sheet, or space webs). The ML analyses contrasted with this in recovering the most likely state for the ancestral mygalomorph as a burrower, and the ancestral atypoid as a burrower with a purse‐web entrance. However, these differences are likely due to the absence of several opportunist, web‐building atypoid taxa from the ML analysis (*Hexurella*, *Mecicobothrium*, and *Megahexura*), and we therefore prefer the hypothesis of the more taxon‐rich MP analysis.

Assuming an opportunist ancestor, obligate burrowing has arisen at least four times independently in the Mygalomorphae: in the Atypoidea (Atypidae and Antrodiaetidae), the Euagridae (some *Cethegus*), the Hexathelidae (*Mediothele*, *Plesiothele*, and some *Scotinoecus* and *Hexathele*), and in the ancestor of the Bipectina (not including Paratropididae). Most of the early branching avicularioid families have opportunistic, web‐building ancestors, however, the ancestral hexathelid was recovered as ambiguous in the MP analysis (which has several additional hexathelid taxa) being either an opportunist with a web entrance or a burrower with an open entrance.

We recovered the ancestor of the Bipectina (‐Paratropididae) as a burrower with a trapdoor entrance, and this behavior was retained in the ancestor of three of the four major bipectine clades: the Venom Clade+, the Domiothelina, and the Theraphosoidina. The ancestor of the Nemesioidina, however, was recovered as a burrower with an open entrance. In the Venom Clade+, burrowing and trapdoor‐building have both been lost in the Atracidae, most of which are opportunists with web entrances (*Atrax* and many *Hadronyche*). In the Domiothelina, the burrowing and trapdoor‐building combination is largely conserved, but the trapdoor has been lost several times independently in favor of an open entrance or another type of entrance modification (collar or turret). Nest‐building has also evolved at least three times independently in the Domiothelina (in the Idiopidae, Halonoproctidae, and Migidae), always from burrowing, trapdoor‐building ancestors, and all nest‐builders retain the trapdoor. This nest‐building + trapdoor niche evolved in the same way in the Theraphosoidina, as in the Barychelidae. Although our analysis includes only a fraction of theraphosid diversity, we recovered the ancestral tarantula as a burrower with an open hole. Finally, in the Nemesioidina, almost the full spectrum of behaviors has evolved from the burrowing + open‐entrance ancestor: trapdoors and other entrance modifications have evolved several times, as has opportunism, and the hypothesized ancestral mygalomorph niche of opportunism + web construction has evolved in the Dipluridae.

Overall, behavioral niche space in the Mygalomorphae can be described in terms of two extremes: at one end are opportunists that build webs at the entrance to the burrow, and at the other are burrowers and nest‐builders that structurally modify their burrow entrance. Intermediate taxa usually burrow, but neither construct a web nor structurally modify their entrance. Shifts across this niche space in both directions have been common in mygalomorph evolution, with almost all major clades including representatives of several/most behavioral niches, despite disparate evolutionary histories (Figure 1).

### 3.2 Variation in somatic morphology and its relationship with behavioral niche

**FIGURE 2:**
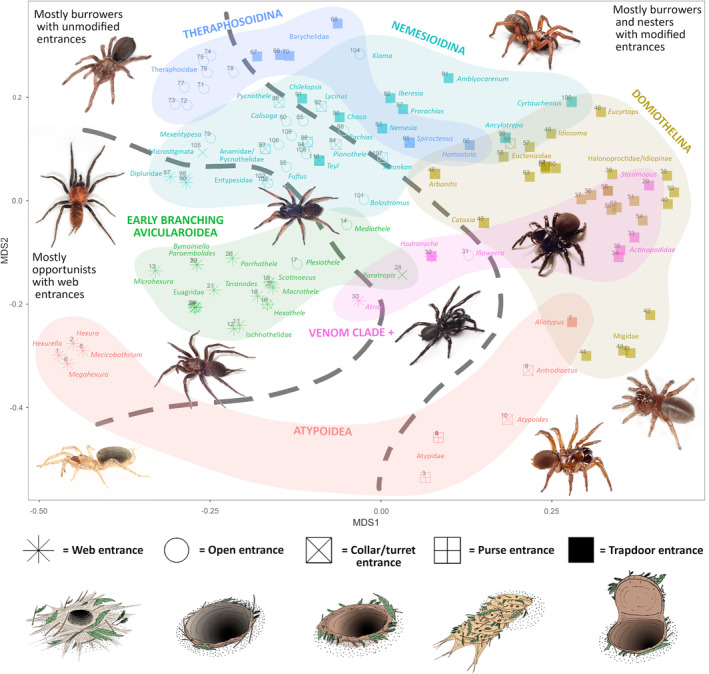
Results of the non‐metric multidimensional scaling analysis (NMDS) of mygalomorph somatic morphology. Colors indicate major phylogenetic clades (corresponding to Figure 1), symbols indicate burrow‐entrance type, and the gray lines roughly divide taxa into opportunists (left), burrowers and non‐burrowers with an open entrance (Centre), and burrowers and nest‐builders with structurally modified burrow entrances (right). Genera and photographers of the habitus shots are as follows (from left to right): *Linothele* (Dipluridae) = M. Ramirez; *Mecicobothrium* (Mecicobothriidae) = N. Ferretti; *Selenocosmia* (Theraphosidae) = J. Wilson; *Namirea* (Euagridae) = J. Wilson; *Namea* (Anamidae) = M. Rix; *Hadronyche* (Atracidae) = E. Yoeman; *Homostola* (Bemmeridae) = J. Bond; *Missulena* (Actinopodidae) = J. Wilson; *Antrodiaetus* (Antrodiaetidae) = J. Bond; and *Calathotarsus* = M. Ramirez. Burrow type illustrations by J. Wilson.

The NMDS ordination shows the heavy influence of behavioral niche on mygalomorph somatic morphology, although evolutionary history also plays a role (Figure 2). A clear behavioral gradient can be seen, with opportunistic, web‐building taxa representing one extreme of the morphological/behavioral spectrum in the bottom‐left of the ordination, and burrowers and nest‐builders with a trapdoor entrance representing the other, on the right. Between these two extremes lies opportunists and burrowers with open entrances (generally clustering slightly left of center), and burrowers with other entrance modifications besides a trapdoor (slightly right of center).

Clearly, many aspects of somatic morphology are strongly influenced by evolutionary history, as many major phylogenetic clades do not overlap, and the proximity of these clades to one another is generally reflective of their phylogenetic relationships (Figure 2). For example, the Atypoidea are at the bottom of the ordination, separate from the Avicularioidea (all other Mygalomorphae), and the Crassitarsi (Nemesioidina + Theraphosoidina) and Venom Clade+ and Domiothelina form clusters. However, many of these clades are spread widely across morpho‐space from left to right, reflecting the diversity of behavioral niches that their species inhabit.

Members of different clades with similar burrowing behaviors are often closer together in morpho‐space than members of the same clade that behave differently, presumably reflecting the convergent evolution of morphological characters that are adapted to particular behavioral niches (e.g., see Table 2). For example, those members of the Antrodiaetidae, Actinopodidae, Stasimopidae, and Bemmeridae that are burrowers with structurally modified burrow entrances all cluster closer to the Domiothelina than to other more closely related taxa that behave differently. Indeed, the position of taxa in morpho‐space often mirrors previous phylogenetic hypotheses based on morphology, for example, *Atrax* (Venom clade), which has independently evolved opportunistic habits and a web entrance, is recovered close to the Hexathelidae, the Actinopodidae (Venom clade) cluster within the Domiothelina, and the bemmerid genera *Spiroctenus* and *Homostola* cluster closest to nemesioid and euctenizid genera, respectively, mirroring their previous taxonomic positions.

**TABLE 2:** Results of the correlation analysis between morphological features (Table 1) and key behavioral traits.

Morphological feature [y]	Behavior [x]	Pairwise comparison analyses		Maximum likelihood (delta‐AICc)			
		PC1	PC2	Indep	x‐dep	y‐dep	Interdep
Elongate posterior lateral spinnerets (C11)	**Web‐building**	**0.016 (6:0)**	**0.016 (6:0:1)**	**11.25**	**0.32**	**0.00**	**2.00**
	Opportunist	0.016 (6:0)	0.031 (5:0:4)	11.32	0.00	2.47	2.28
	Burrowing	0.016 (0:6)	0.031 (0:5:7)	10.89	0.00	6.06	2.07
	Entrance modification	0.03 (0:5)	0.063 (0:4:10)	21.20	0.00	13.00	2.04
	Trapdoor entrance	0.125 (0:3)	0.5 (0:1:9)	–	–	–	–
Widely separated spinneret pairs (C2)	**Web‐building**	**0.016 (6:0)**	**0.016 (6:0:1)**	**11.25**	**0.32**	**0.00**	**2.00**
	Opportunist	0.016 (6:0)	0.031 (5:0:5)	11.32	0.00	2.47	2.28
	Burrowing	0.016 (0:6)	0.06 (0:4:9)	10.89	0.00	6.06	2.07
	Entrance modification	0.03 (0:5)	0.13 (0:3:11)	11.16	0.00	13.00	2.04
	Trapdoor entrance	0.13 (0:3)	0.5 (0:1:9)	–	–	–	–
Pseudo‐segmented apical segment of posterior lateral spinnerets (C10)	Web‐building	0.063 (4:0)	0.063 (4:0:4)	–	–	–	–
	Opportunist	0.063 (4:0)	0.063 (4:0:7)	–	–	–	–
	Burrowing	0.063 (0:4)	0.063 (0:4:10)	–	–	–	–
	Entrance modification	0.063 (0:4)	0.063 (0:4:10)	–	–	–	–
	Trapdoor entrance	0.25 (0:2)	0.5 (0:1:9)	–	–	–	–
Short apical segment of posterior lateral spinnerets (C9)	Web‐building	0.125 (0:3)	0.5 (0:1:5)	–	–	–	–
	Opportunist	0.03 (0:5)	0.25 (0:2:7)	15.94	3.08	0.96	0.00
	Burrowing	0.34 (4:2)	0.31 (3:1:8)	–	–	–	–
	**Entrance modification**	**0.008 (7:0)**	**0.016 (6:0:5)**	**12.63**	**4.02**	**11.96**	**0.00**
	Trapdoor‐building	0.008 (7:0)	0.031 (5:0:4)	10.76	5.21	0.00	1.55
Presence of a rastellum (C51)	Web‐building	0.03 (0:5)	0.13 (0:3:4)	11.07	7.56	0.00	2.25
	Opportunist	0.008 (0:7)	0.063 (0:4:6)	11.74	4.18	0.00	1.52
	**Burrowing**	**0.004 (8:0)**	**0.016 (6:0:6)**	**10.56**	**2.60**	**0.00**	**1.29**
	Entrance modification	0.01 (9:1)	0.0078 (7:0:5)	10.89	1.29	10.21	0.00
	Trapdoor‐building	0.035 (7:1)	0.063 (5:0:5)	10.75	0.53	7.36	0.00
Presence of a serrula (C43)	Web‐building	0.016 (6:0)	0.063(4:0:4)	11.66	5.30	0.00	2.05
	Opportunist	0.004 (8:0)	0.016 (6:0:5)	11.72	1.60	0.00	2.10
	**Burrowing**	**0.002 (0:9)**	**0.004 (0:8:6)**	**10.80**	**0.02**	**5.05**	**0.00**
	Entrance modification	0.008 (0:7)	0.008 (0:7:6)	12.86	0.00	1.65	0.93
	Trapdoor‐building	0.008 (0:7)	0.25 (0:2:8)	9.53	9.08	0.04	0.00
Digging spines on legs I–II (C18)	Web‐building	0.03 (0:5)	0.125 (0:3:5)	7.96	5.26	0.00	2.14
	Opportunist	0.03 (0:5)	0.125 (0:3:8)	7.68	0.56	0.00	1.43
	Burrowing	0.11 (5:1)	0.063 (4:0:10)	–	–	–	–
	**Entrance modification**	**0.03 (5:0)**	**0.063 (4:0:10)**	**8.93**	**3.09**	**0.00**	**1.49**
	Trapdoor‐building	0.063 (4:0)	0.5 (1:0:9)	–	–	–	–
Presence of scopulae on the anterior tarsi/metatarsi (C20)	Web‐building	0.5 (1:2)	0.5 (1:2:5)	–	–	–	–
	Opportunist	0.5 (1:2)	0.5 (1:2:8)	–	–	–	–
	Burrowing	0.5 (2:1)	0.25 (2:0:12)	–	–	–	–
	Entrance modification	0.125 (3:0)	0.5 (1:0:13)	–	–	–	–
	Trapdoor‐building	0.31 (3:1)	0.5 (1:0:9)	–	–	–	–
Leg III being thicker and at least as long as leg II (C13)	Web‐building	0.016 (0:6)	0.063 (0:4:4)	11.42	8.80	0.00	2.21
	Opportunist	0.004 (0:8)	0.063 (0:4:7)	11.58	4.71	0.00	2.00
	Burrowing	0.002 (9:0)	0.031 (5:0:8)	9.98	0.00	0.56	0.05
	**Entrance modification**	**0.004 (8:0)**	**0.0039 (8:0:5)**	**12.01**	**1.35**	**0.00**	**1.22**
	Trapdoor‐building	0.109 (5:1)	0.063 (4:0:4)	–	–	–	–
Spines of leg III mostly dorsal (C14)	Web‐building	0.016 (0:6)	0.063 (0:4:4)	7.32	3.86	1.00	0.00
	Opportunist	0.004 (0:8)	0.031 (0:5:6)	10.34	2.47	3.03	0.00
	**Burrowing**	**0.002 (9:0)**	**0.016 (6:0:8)**	**8.51**	**0.00**	**5.48**	**0.69**
	Entrance modification	0.063 (6:1)	0.031 (5:0:9)	9.53	0.00	3.38	1.94
	Trapdoor‐building	0.23 (5:2)	0.19 (4:1:3)	–	–	–	–
Patella III with pro‐dorsal patch of >3 thorn‐like setae (C15)	Web‐building	0.016 (0:6)	0.063 (0:4:3)	8.53	9.09	0.00	2.21
	Opportunist	0.008 (0:7)	0.063 (0:4:6)	9.81	3.95	0.00	1.48
	**Burrowing**	**0.004 (8:0)**	**0.031 (5:0:7)**	**9.59**	**0.00**	**1.96**	**0.28**
	Entrance modification	0.109 (5:1)	0.031 (5:0:8)	10.34	0.00	5.72	2.07
	Trapdoor‐building	0.69 (2:2)	0.75 (1:1:7)	–	–	–	–
Presence of a common tubercle (C35)	Web‐building	0.03 (5:0)	0.25 (2:0:6)	7.89	9.55	0.00	0.85
	**Opportunist**	**0.008 (7:0)**	**0.5 (2:1:8)**	**9.73**	**9.65**	**0.00**	**1.82**
	Burrowing	0.036 (1:7)	0.13 (0:3:11)	8.97	8.15	0.00	0.76
	Entrance modification	0.063 (1:6)	0.31 (1:3:10)	–	–	–	–
	Trapdoor‐building	0.11 (1:5)	1 (0:0:10)	–	–	–	–
A compact, rectangular eye group (C32–34)	Web‐building	0.063 (4:0)	0.5 (1:0:7)	–	–	–	–
	Opportunist	0.03 (5:0)	0.5 (1:0:10)	5.28	4.02	1.37	0.00
	Burrowing	0.34 (2:4)	0.25 (2:0:12)	–	–	–	–
	**Entrance modification**	**0.03 (0:5)**	**0.06 (0:4:10)**	**6.58**	**0.00**	**1.98**	**0.99**
	Trapdoor‐building	0.19 (1:4)	0.25 (2:0:8)	–	–	–	–
A wide‐eye group (C22)	Web‐building	0.063 (0:4)	1 (0:0:8)	–	–	–	–
	Opportunist	0.063 (0:4)	1 (0:0:11)	–	–	–	–
	Burrowing	0.19 (4:1)	0.5 (1:0:13)	–	–	–	–
	Entrance modification	0.063 (4:0)	0.25 (2:0:12)	–	–	–	–
	Trapdoor‐building	0.31 (3:1)	0.5 (1:0:9)	–	–	–	–
Anterior lateral eyes in an advanced position relative to anterior median eyes (C23)	Web‐building	0.25 (0:2)	1 (0:0:8)	–	–	–	–
	Opportunist	0.25 (0:2)	1 (0:0:11)	–	–	–	–
	Burrowing	0.75 (1:1)	1 (0:0:14)	–	–	–	–
	Entrance modification	0.25 (2:0)	0.5 (1:0:13)	–	–	–	–
	Trapdoor‐building	0.25 (2:0)	0.5 (1:0:9)	–	–	–	–

*Note*: Significant positive correlations are indicated in green, negative in red, and the behavior(s) most strongly correlated with a morphological feature is in bold. Results of PC1 follow the format: *p*‐value (positive pairs: negative pairs). Results of PC2 follow the format: *p*‐value (positive pairs: negative pairs: neutral pairs). Positive pairs represent phylogenetically independent pairs of taxa that contrast in both the morphological feature and the behavior in a pattern indicating paired loss or paired gain of this feature and behavior. Negative pairs show the opposite pattern, indicating that when one character is lost the other is gained, or vice versa. In neutral pairs, the phylogenetically independent taxa vary in just one of the two characters (neutral pairs are not included in PC1). In the ML analysis, a delta‐AICc of 0 indicates the best‐fitting model for that hypothesis, and in alternate models, the larger the delta‐AICc value, the worse that model performed relative to the best model.

### 3.3 Correlated evolution of morphology and behavior

**FIGURE 3:**
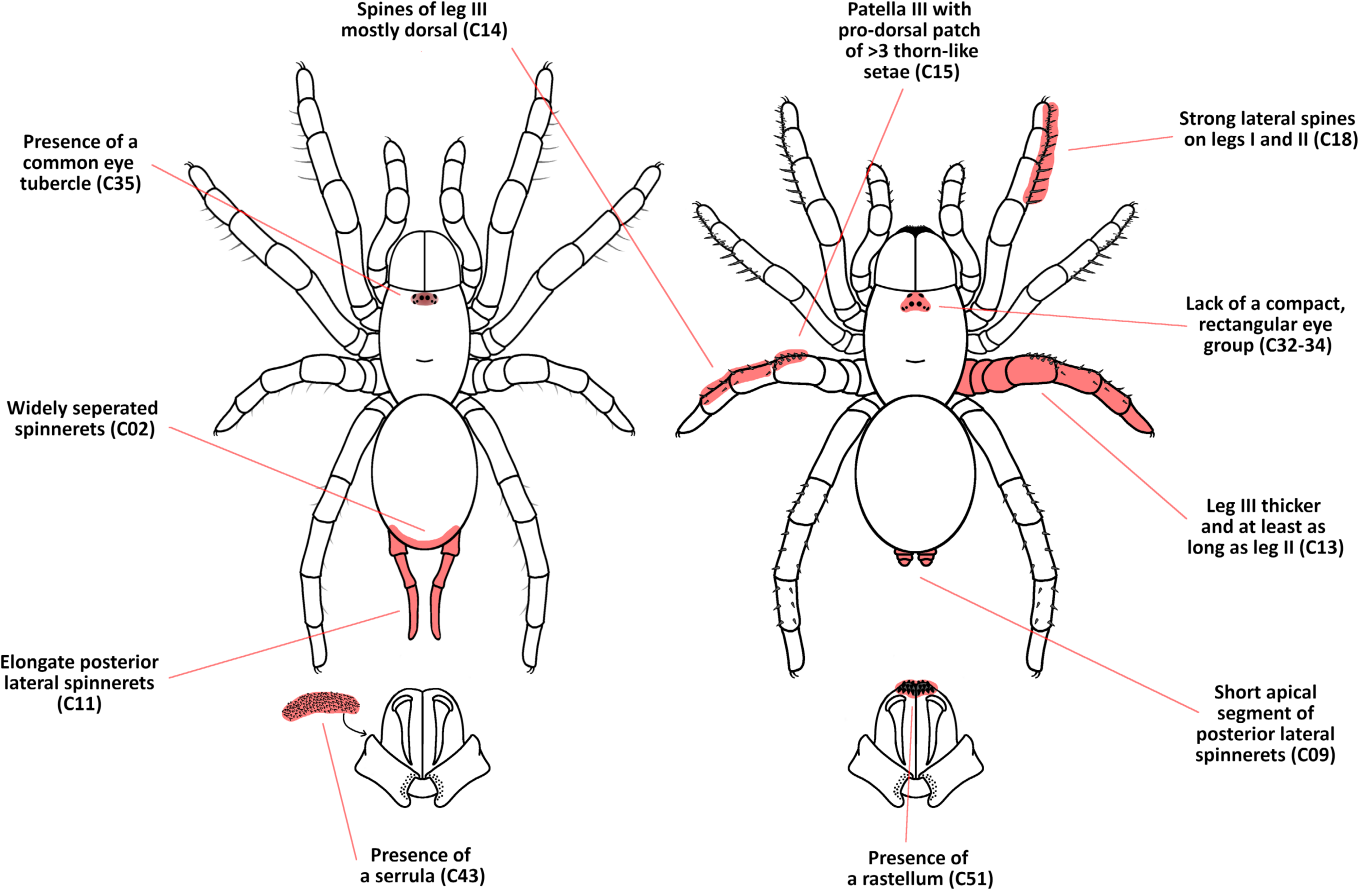
Schematic representations of somatic morphology at each extreme of the mygalomorph adaptive landscape: An opportunist with a web entrance (left) and a burrower/nest‐builder with a structurally modified burrow entrance (right). Red highlights and labels are representations of the 11 morphological features found to be correlated with key behaviors (see Table 2 for specifics of correlation).

Of the morphological features that we tested for correlation with behavior (see Table 1), we identified significant patterns of correlation in 11 (Table 2, Figure 3). Analyses using pairwise comparisons (PC) and maximum likelihood (ML) were largely corroborative, with strongest hypotheses of correlation returning the strongest significance values in the PC analyses, and very high delta‐AICc values for the uncorrelated (independent) model in the ML analysis, indicating the poor fit of this model relative to the best correlated (dependent) mode. For characters analyzed using ML, the uncorrelated model was almost always the worst performing (with the highest delta‐AICc), and delta‐AICc values were usually low for all dependent models, signifying little difference in model fit between different dynamics of dependence.

Patterns of correlation between the spinnerets and behavior were as expected: elongate posterior lateral spinnerets and widely spaced spinnerets were strongly positively correlated with the construction of a capture web at the retreat entrance and to a lesser extent with opportunist retreat construction (almost all web‐builders are opportunists), and were negatively correlated with burrowing and entrance modification. In contrast, short apical segments of the posterior lateral spinnerets were most strongly correlated with structural modification to the burrow entrance and negatively correlated with opportunism and web‐building (opportunists virtually never structurally modify their retreat entrance). The rastellum returned strong positive correlation with both burrowing and entrance modification, however, the second pairwise comparison analysis (PC2) revealed many cases of “neutral change” with respect to the rastellum, meaning that these characters and behaviors have sometimes evolved independently from each other over the mygalomorph evolutionary tree. The serrula returned the opposite pattern to the rastellum, being positively correlated with web‐building and opportunism, and negatively correlated with burrowing and entrance modification, with negative correlation with burrowing returning the strongest correlation. “Digging spines” did not return a significant correlation with burrowing, but instead with entrance modification (positive). The presence of scopulae showed no correlation with any of the behaviors tested. All modifications to the posterior legs – enlargement relative to the anterior legs, dorsal bias in macrosetation, and presence of a thorn patch on prodorsal patella III – showed a strong positive correlation with both burrowing and burrow‐entrance modification. Finally, the presence of a common eye tubercle was positively correlated with opportunistic burrowing (although there are many cases of neutral change, see PC2), and a compact rectangular eye group was negatively correlated with burrow‐entrance modification; however, the two specific modifications to the eye group which were tested for correlation (widening of the eye group and anteriorly positioned anterior lateral eyes) did not return significant correlation, despite each only occurring in taxa with modified burrow entrances.

## 4 DISCUSSION

Morphological convergence is often viewed as a “remarkable” phenomenon, yet it is ubiquitous across the tree of life, and some believe that understanding why represents one of biology's most pressing questions (Conway Morris, 2010; Losos, 2011). However, the causes of convergence can be nuanced, and their identification requires a thorough understanding of the function of morphological features (Losos, 2011). In spiders, recent advances in our understanding of phylogenetic relationships (e.g., Kulkarni et al., 2021; Opatova et al., 2020; Wheeler et al., 2017) have led to renewed interest in convergence, with several recent analyses focusing on the convergence of particular structures (e.g., Ramírez et al., 2021) and behaviors (e.g., Kallal et al., 2020; Wolff et al., 2019). However, the influence of convergence on mygalomorph spiders has not previously been explored analytically.

Our results reveal that the convergent evolution of phenotype in correlation with behavioral niche is a pervasive trend in the evolution of mygalomorph spiders. Their adaptive landscape is simple and constrained at two extremes: at one end are opportunistic taxa that inhabit existing spaces and construct capture webs, and at the other are taxa that construct their own burrow or nest, and structurally modify the entrance, for example, with a trapdoor (Figure 2). A spectrum exists between these extremes, but most intermediate taxa still burrow, or show facultative burrowing habits, but do not structurally modify the entrance. Within these constraints, changes in the niche occupied have been common in the evolution of the infraorder, and have occurred in both directions (Figure 1). For example, the general trend in both the Atypoidea and Avicularioidea is that burrowing, trapdoor‐building taxa have evolved from opportunistic, web‐building ancestors, yet in (at least) the Venom clade and the Nemesioidea, the opportunistic, web‐building niche has evolved again, independently (Figure 1). Repeated evolution of morphological traits associated with different behavioral strategies in this adaptive landscape is one of the primary forces shaping somatic morphology in the Mygalomorphae, and this trend is clear in both overall morphology (Figure 2) and in those morphological features that are intuitively adaptive (Tables 1 and 2; Figure 3). Convergent characters generally represent changes in the number, shape, size, or position of pre‐existing structures, rather than the gain and loss of complex structures or systems, and this may explain the evolutionary plasticity of these characters. The historical use of these characters to infer phylogenetic relationships explains, at least in part, the conflict between traditional morphological hypotheses and new molecular ones. Indeed, it is now clear that the “Dipluridae” sensu *lato* and the previous higher classification “Rastelloidina” are both artificial groups lumping together taxa from either end of the mygalomorph adaptive landscape (Raven, 1985).

### 4.1 Insights into the function of convergent morphological features

This study is the first to quantify the strong correlation between behavioral niches and a suite of convergent morphological features within the Mygalomorphae. In particular, features of the spinnerets, leg chaetotaxy, and eye group, as well as the rastellum and serrula, exhibit strong patterns of correlation with behavior, and an examination of their likely function provides insights into the potential drivers of convergent evolution within the group.

#### 4.1.1 Spinnerets

Elongate, widely spaced posterior lateral spinnerets are correlated with web‐building (Table 2; Figure 3). Their length presumably allows for the efficient application of wide swathes of silk during the construction and repair of capture webs, as has been observed in *Linothele* (Eberhard & Hazzi, 2013; Nicolás Paz, 1988). Their widely separated position likely also aids in the independent, unilateral, or asymmetrical use of each spinneret during web construction, for example, during the attachment of individual silk sheets (as observed in *Linothele macrothelifera*; Eberhard & Hazzi, 2013). In contrast, very short apical segments of the PLS (and short spinnerets in general) are correlated with structural modification in the retreat entrance (Table 2; Figure 3) and are probably better for the precise application of strong, thin bands of silk (as observed in *Ummidia*: Coyle, 1981). The precise application of silk may be important for the integrity of these entrance structures, for example, in the construction of a trapdoor hinge, or the substrate/silk matrix of a trapdoor or turret (Coyle, 1981; Coyle et al., 1992). During burrow and burrow‐entrance construction, these short spinnerets have been observed to work together synchronously and/or rhythmically, usually applying silk to the same area, explaining their position close together on the abdomen in these species (Coyle et al., 1992; Mayo, 1988).

#### 4.1.2 Rastellum and serrula

The rastellum is strongly correlated with both burrowing and door construction (Table 2; Figure 3). Observations of burrowing taxa indicate that it is used for compaction of the burrow shaft and entrance structures (Coyle, 1981; Coyle et al., 1992) plus excavation (Gertsch, 1949; Nascimento et al., 2021). However, both burrowing and entrance modification occur in taxa that do not possess a rastellum (e.g., Theraphosidae and Migidae, respectively), suggesting that other factors may also influence whether the structure is necessary, for example, the substrate in which the spider burrows. The function of the serrula in spiders is generally assumed to involve manipulation of prey items (Jocqué & Dippenaar‐Schoeman, 2006). We found that it was positively correlated with opportunistic retreats, and negatively correlated with burrowing (Table 2; Figure 3). The functional reasons for this are unclear, although a speculative explanation for the negative correlation of the serrula with burrowing could be a tendency for it to become clogged with substrate while burrowing, because substrate is carried using the chelicerae/pedipalps during burrow construction, and so would likely come into contact with the serrula (Coyle, 1974, 1981; Mayo, 1988).

#### 4.1.3 Leg chaetotaxy

Surprisingly, the so‐called “digging spines” – strong lateral spines on the anterior legs and pedipalps, did not show a positive correlation with digging, but only with burrow‐entrance modification (Table 2; Figure 3). That digging is not the primary role of these spines is supported by behavioral studies of burrowing taxa that observed that the chelicerae and fangs are used for substrate excavation, not the legs (Coyle, 1981; Coyle et al., 1992; Mayo, 1988; Nascimento et al., 2021). Furthermore, some taxa that do not burrow (e.g., many Migidae) still possess these spines, although they have lost other features associated with burrowing (e.g., pro‐dorsal spine patches on patella III). We suggest that these spines function primarily during prey capture in species with modified burrow entrances, which tend to have smaller foraging areas (Main, 1982) and hunt by lunging from the burrow entrance and restraining prey with the anterior legs and pedipalps (Coyle, 1981, 1986; Hils & Hembree, 2015). Although no correlation was found between scopulae and behavior, in taxa that modify the burrow entrance scopulae clearly replace the function of digging spines because the only entrance‐modifying taxa without digging spines possess scopulae, adding to the well‐supported hypothesis that a function of both structures is to restrain prey (e.g., see Eggs et al., 2015; Pekár et al., 2011; Wolff & Gorb, 2016).

Enlarged posterior legs, a dorsal bias in spine position on the posterior legs, and the presence of pro‐dorsal thorn patches on patella III are all correlated with both burrowing and burrow entrance modification (Table 2; Figure 3). Behavioral studies on several burrowing species indicate that the posterior legs are braced against the burrow wall to anchor the spider (Bond & Coyle, 1995; Coyle, 1981; Decae & Bosmans, 2014; Hils & Hembree, 2015). This is done during routine movement, but also serves a defensive function in species that hold their burrow entrance shut when disturbed. Larger, stronger posterior legs and dorsal spines likely enhance this bracing function.

#### 4.1.4 Eye group

The eye tubercle was found to be positively correlated with opportunistic burrowing, and a standard, compact, rectangular eye group was found to be negatively correlated with burrow entrance modification (indicating that change from this state generally occurs in taxa with modified entrances; Table 2; Figure 3). It seems most probable that these changes in the eye group relate to the amount and direction of light exposure (and therefore visual information) in different retreat types, for example, almost all opportunist taxa have relatively open retreat entrances, and when foraging at the retreat entrance, would be exposed to light from all directions. In contrast, burrowing taxa with modified entrances would be exposed to light from only one direction (the entrance), and far less light in general. This is, however, in contrast to several previous studies which indicate that vision is not important for foraging in a range of mygalomorph species (see Coyle, 1986, for a list of relevant literature). An alternative to this is that changes in eye group shape relate to carapace shape, which itself is reflective of different behavioral niches.

### 4.2 Niche dynamics within the Mygalomorphae

That niche evolution has occurred in both directions several times across the mygalomorph adaptive landscape (Figures 1 and 2) indicates that the “optimal” niche changes depending on environmental conditions due to trade‐offs in niche dynamics (Winemiller et al., 2015). Some potential aspects that show patterns of variation across the adaptive landscape include prey‐capture area and method, predator defense, microhabitat, and microclimate regulation (see specific references below).

If we consider the two extremes of the mygalomorph adaptive landscape, we see strategies that vary across all four of the dimensions mentioned above. Mygalomorph spiders rely heavily on substrate‐borne vibrations to detect prey, and their silken constructions (and the objects directly attached to them) determine the size of their foraging area (Coyle, 1986; Main, 1982). Opportunistic, web‐building taxa have extensive prey‐capture areas because they detect prey across the entire capture web, which also helps to slow/entangle prey, decreasing the spider's need to physically restrain it (Coyle, 1986, 1995). Web‐building taxa construct no clearly defensive structures except for the web itself and tend to escape disturbance by retreating up fissures in the substrate (JDW, personal observation), thus taking advantage of the complex microhabitats in which they live, which must have adequate crevices under rocks, in or around vegetation, or under embankments for retreat construction (Coyle, 1995; Eberhard & Hazzi, 2013; Raven, 1983). As these spiders generally do not burrow, they probably have less ability to regulate the microclimate of their retreat and less protection against natural disasters such as floods, although the retreats of some species will follow natural crevices deep into embankments or under rocks, which may serve a similar regulatory function to a burrow and explain the occurrence of some opportunistic, web‐building taxa in quite arid environments (e.g., *Cethegus* in Australia, Raven, 1983; *Euagrus* in North and Central America, Coyle, 1988).

At the other end of the spectrum are burrowing and/or nesting taxa that modify their entrance with a trapdoor. Observations suggest that some trapdoor spiders will not strike at prey unless it touches the burrow entrance or comes within millimeters of it, indicating a comparatively tiny foraging area (Bond & Coyle, 1995; Coyle et al., 1992). Within this tiny foraging area, they rely entirely on physicality and the element of surprise to restrain prey, and this probably explains adaptations such as the strong lateral spines found in many species with trapdoors or other entrance modifications. Further evidence that a trapdoor entrance reduces foraging area is provided by the multitude of modifications that trapdoor‐building species construct to extend their sensory radius, including radiating silk‐ or twig‐lines (Main, 1957; Rix, Cooper, et al., 2017; Rix, Raven, et al., 2017), soil tabs (Coyle & Icenogle, 1994), and foliage “mustaches” (Rix, Cooper, et al., 2017; Rix, Raven, et al., 2017) among others (Coyle, 1986). Open burrows and/or burrows with other types of modification besides a trapdoor probably increase the prey‐capture radius relative to a trapdoor entrance, as evidenced by Coyle (1986), who demonstrated that collar‐building *Antrodiaetus* enjoy a larger prey‐capture area than trapdoor‐building *Aliatypus* (both family Antrodiaetidae), primarily because strikes in the “dorsal sector” are restricted in the latter by the trapdoor hinge. Regarding predator/parasite defense, the burrow is a double‐edged sword, providing both camouflage and a means of protection, but also limiting avenues of escape. Certain fungi, buthid scorpions, pompilid wasps, and acrocerid flies are known to specialize on burrowing mygalomorph spiders (Kurczewski et al., 2021; Pérez‐Miles & Perafán, 2017), and predators such as centipedes (MGR, personal observation) and even other araneophagic spiders may target them (Dippenaar‐Schoeman, 2002). This has led to the evolution of myriad defensive strategies in burrowing taxa, including secondary escape shafts (Harvey et al., 2018), false bottoms (Main, 1985), spherical pellets used to block the entrance (Leroy & Leroy, 2005), phragmotic abdomens (Rix et al., 2018), urticating setae (Bertani & Guadanucci, 2013), and of course, entrance modifications which camouflage the burrow and can be held closed against intruders. Finally, the construction of a burrow allows access to relatively bare habitats without natural crevices, and may also allow greater regulation of the microclimate in the burrow (primarily temperature and humidity), and resistance to natural disasters like droughts and floods (Cloudsley‐Thompson, 1983; Coyle, 1986). This regulatory function may be further increased by modifications that allow the burrow entrance to be closed, for example, a trapdoor, which may explain why, in families containing both trapdoor‐builders and species that utilize a more open entrance type, the trapdoor‐builders are often those that have spread into arid environments (e.g., in the Australian Idiopidae, Rix, Cooper, et al., 2017; Rix, Raven, et al., 2017), and the North American Euctenizid genera *Apomastus* and *Aptostichus* (Bond, 2004, 2012). However, there are also burrowing species with an open entrance that have adapted and radiated in arid environments (e.g., the theraphosid genus *Aphonopelma*, Hamilton et al., 2011, and the anamid genus *Aname*, Rix et al., 2021), and direct experiments on a trapdoor‐building lycosid found that the trapdoor provides negligible difference to conditions at the bottom of the burrow, indicating that it may primarily serve other functions such as predator defense or flood avoidance (Steves et al., 2021).

The evolution of nest retreats deserves specific discussion. Our results indicate that nests have always evolved from burrowing, trapdoor‐building ancestors. As nests are short and presumably less well‐insulated than a burrow, these taxa probably lose some degree of microclimate regulation, which explains why most nest‐building taxa occur in mesic environments (e.g., Migidae, Griswold & Ledford, 2001, *Sason*, Raven, 1986). However, Coyle (1986) points out a likely benefit of nesting, which is that the spider can sense prey over the entire exposed surface of the nest, expanding the foraging area relative to a burrow. Many nests have two trapdoor entrances, one at each end, and this probably allows greater exploitation of this expanded prey‐capture area and provides a second escape route from predators. Nests also allow the exploitation of new microhabitats, as they are often constructed off the ground, on tree trunks, or on cave walls (Decae et al., 2021; Griswold & Ledford, 2001; Raven, 1986). In this way, evolution from a burrow to a nest could represent an evolutionary pathway with similar trade‐offs to the opportunistic, web‐building niche: the sacrifice of microclimate regulation for an expanded foraging area and exploitation of a different microhabitat.

Patterns of niche trade‐offs in the Mygalomorphae are clearly complex and cannot be explained with reference to a single environmental variable. Climate and weather, environmental complexity and niche availability, and the abundance of predators and prey probably all play a role in determining the success of a particular behavioral niche in an environment, and the changes in these factors over deep time probably contributed to the dynamic evolution of behavior in the group. Furthermore, microhabitat differences mean that in optimal conditions, species inhabiting different niches often occur together, for example, in sub‐tropical eastern Australia, many areas exist where several burrowing (e.g., Idiopidae, Anamidae), nesting (Barychelidae, Migidae), and opportunistic (Euagridae, Hexathelidae, and Atracidae) taxa occur in direct sympatry. In general, burrowing taxa probably have the highest resilience to environmental extremes and are also able to exploit relatively bare microhabitats. In contrast, web‐building and nest‐building taxa probably require milder environmental conditions but allow the spider to expand its foraging area and exploit new microhabitats: existing spaces under logs, embankments and foliage for opportunists, and hard substrates off the ground for nest‐builders.

### 4.3 Constraints on the mygalomorph evolutionary landscape

Despite differences in the niche dimensions mentioned above, overall, mygalomorph life histories are remarkably homogeneous: all are long‐lived, sedentary spiders that live in permanent retreats on or within the substrate or foliage (Raven, 1985). Because extant members of the suborder Mesothelae also live this way, it is often assumed to represent the ancestral life history of extant spiders. In contrast, the Araneomorphae occupy an incredibly diverse array of niches, and include aerial web builders, burrowers, cursorial hunters, and ambush specialists living in all types of microhabitats both on and off the ground (Foelix, 1996). We can, therefore, gain insight into the constraints on the mygalomorph adaptive landscape by understanding how the Araneomorphae have broken free from it.

Key morphological innovations allowing the Araneomorphae to inhabit new niche space were probably the piriform + ampullate gland spigot system (P + A system) and tracheal posterior respiratory systems (Levi, 1967; Ramírez et al., 2021). The P + A system allows the attachment of individual silk strands to the substrate or each other and is crucial for the use of drag lines and the construction of complex silk structures away from the substrate, such as aerial webs (Coddington & Levi, 1991; Ramírez et al., 2021; Wolff et al., 2019). It is present in almost all araneomorph spiders, and ancestral state reconstructions have now confirmed its origins in the ancestor of the group (Ramírez et al., 2021). Silk glands and spigots of the Mygalomorphae deserve more attention, but presently, no mygalomorph is known to possess an equivalent silk‐attachment system (Palmer, 1991). This probably means that, despite their extensive use of silk, they cannot create complex, load‐bearing silk structures away from the substrate.

Tracheal respiratory systems, which have only evolved in the Araneomorphae, allow oxygen to be directed to muscles where it is needed most, facilitating localized, energy‐demanding activities (Levi, 1967; Ramírez et al., 2021). In their recent study of respiratory system evolution in spiders, Ramírez et al. (2021) showed that tracheal systems evolved several times independently and proposed that their original benefit was directing oxygen to the spinneret muscles to facilitate the new, energy‐expensive spinning procedures associated with the P + A system. Tracheal systems have, however, been co‐opted to direct oxygen into the prosoma in highly active, hunting groups such as the Dionycha (Ramírez et al., 2021). Because of their small spiracle openings, tracheal systems probably also reduce susceptibility to desiccation and are therefore likely to be adaptive in active, cursorial niches, especially in small spiders (Levi, 1967). Mygalomorphae possess the symplesiomorphic posterior respiratory system consisting of a pair of book lungs. These allow only localized oxygen exchange and have larger more exposed openings, and this is probably a major constraint limiting the evolution of active, cursorial niches in the Mygalomorphae.

A final consideration is the ecological constraint of niche availability. Both the aerial web‐building niche and active, cursorial niches were inhabited early in araneomorph evolution (Kallal et al., 2020), and therefore opportunity for mygalomorph ancestors to exploit these niches would have been limited by direct competition with their araneomorph relatives. The mygalomorph adaptive landscape is narrow, but they are well‐adapted to their sedentary lifestyle. The substrate‐bound, retreat‐building niche has re‐evolved in many araneomorph families (e.g., members of the Segestriidae, Filistatidae, Eresidae, Zodariidae, Udubidae, Lycosidae, and Sparassidae), yet the Mygalomorphae must be thought of as the masters of this niche space, having remained a major faunal component within it for over 350 million years (Opatova et al., 2020).

## AUTHOR CONTRIBUTIONS

**Jeremy D. Wilson:** Conceptualization (lead); data curation (lead); formal analysis (lead); funding acquisition (equal); investigation (lead); methodology (lead); visualization (lead); writing – original draft (lead). **Jason E. Bond:** Conceptualization (equal); data curation (equal); funding acquisition (equal); supervision (equal); writing – review and editing (equal). **Mark S. Harvey:** Conceptualization (supporting); data curation (supporting); funding acquisition (equal); supervision (equal); writing – review and editing (equal). **Martín J. Ramírez:** Conceptualization (equal); data curation (equal); funding acquisition (equal); supervision (equal); writing – review and editing (equal). **Michael G. Rix:** Conceptualization (equal); data curation (equal); funding acquisition (equal); supervision (equal); writing – review and editing (equal).

## CONFLICT OF INTEREST

The authors declare no conflicting interests.

## Supporting information


Appendices S1–S3

## Data Availability

The morphological dataset and exemplar specimen information are available in Dryad (Wilson, Bond, et al., 2022): https://doi.org/10.5061/dryad.547d7wmcm.
