# Supplementary material for: Correlation with a limited set of behavioral niches explains the convergence of somatic morphology in mygalomorph spiders
Source: Ecol Evol. 2023 Jan 9;13(1):e9706. doi: 10.1002/ece3.9706 (PMC9830016; doi:10.1002/ece3.9706)
Supplement: Supplementary file 1 — Appendices S1–S3 [file ECE3-13-e9706-s001.docx]

**APPENDIX S1: SUPPLEMENTARY FIGURE**

**
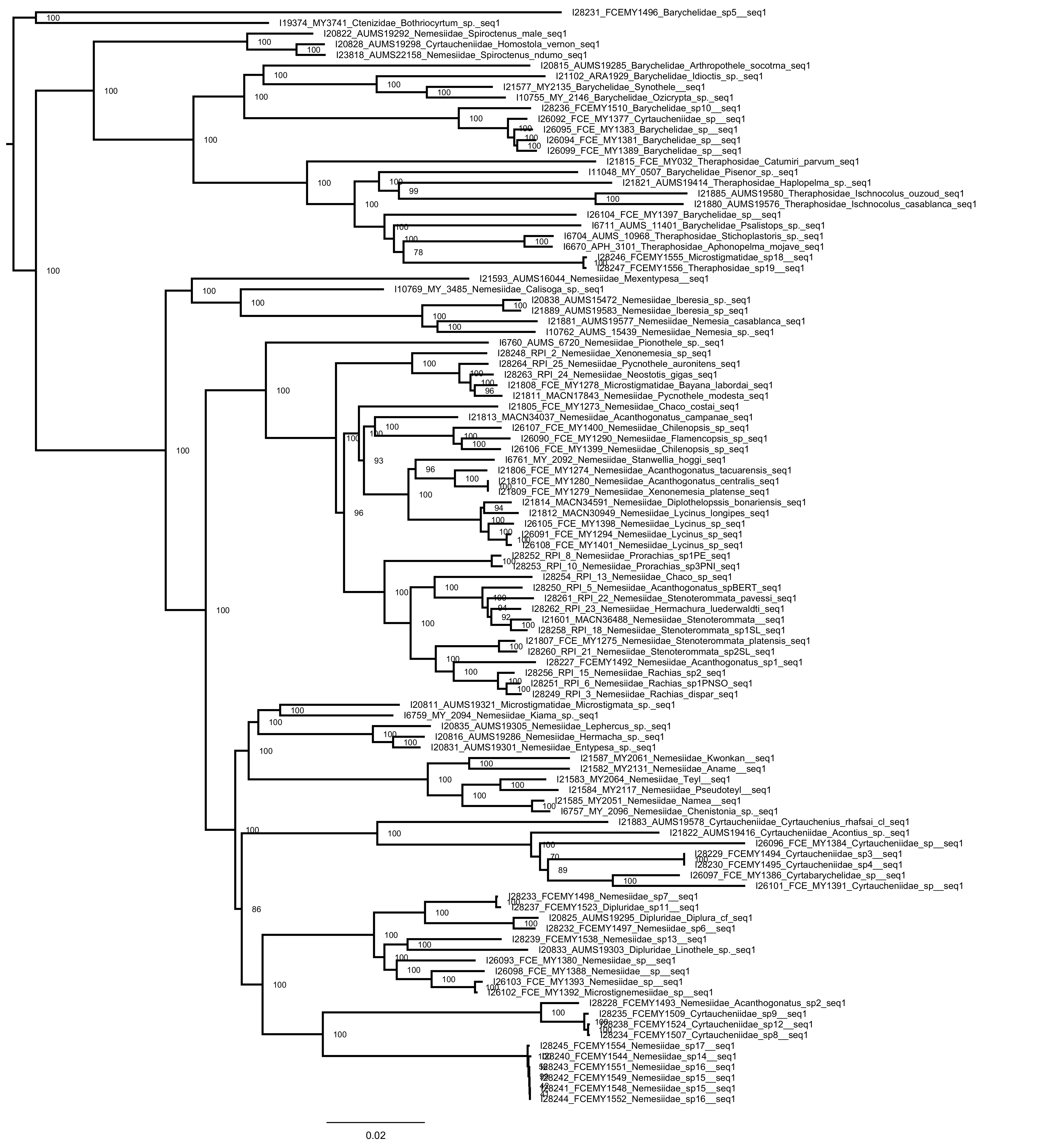
**

**Figure 4. Maximum likelihood tree generated using IQtree (Nguyen et al., 2015) with the alignment and partition files of Montes de Oca et al. (2022). This was then used as one of the input trees for supertree construction.**

**APPENDIX S2: MORPHOLOGICAL CHARACTERS AND STATES**

1. ***Spinning field, position relative to anal tubercle:*** *widely separated = 0; close = 1.*
2. ***Spinnerets, degree of separation:*** *close, distance between PMS bases less than or roughly even with width of anal tubercle = 0; separated, distance between PMS distinctly wider than width of anal tubercle = 1.*

**Remarks:** We differ from past analyses by discretizing this character based specifically on the distance between the PMS relative to the anal tubercle, to decrease ambiguity between states.

1. ***Anterior lateral spinnerets:*** *absent = 0; present = 1.*
2. ***Anterior lateral spinnerets, number of segments:*** *two = 0; one = 1.*
3. ***Anterior lateral spinnerets, size relative to posterior median spinnerets:*** *smaller = 0; sub-equal = 1; larger = 2.*
4. ***Posterior median spinnerets:*** *absent = 0; present = 1.*
5. ***Posterior median spinnerets, shape:*** *digitiform = 0; wide, obliquely triangular tips = 1.*
6. ***Posterior median spinnerets, australotheline crescent:*** *absent = 0; present = 1.*

**Remarks:** The australotheline crescent is a crescent of hard cuticle present in the soft, flexible tissue at the base of the posterior median spinnerets, present in the Australian euagrinae and *Masteria*.

1. ***Posterior lateral spinnerets, apical segment, shape:*** *domed/triangular (length <1.5x width) = 0; digitiform (length > 1.5–3x width) = 1.*

**Remarks:** We differ from past analyses by discretizing this character based specifically on the length/width ratio of the segment to decrease ambiguity between states. Some previous researchers have separated “domed” and “triangular” spinnerets however we found the boundary between these states ambiguous. If a genus is scored as polymorphic, that indicates that it either includes species that fall into each state (e.g., *Aliatypus*), or that it falls on the threshold between states (e.g., *Illawarra*).

1. ***Posterior lateral spinnerets, apical segment, pseudosegmentation:*** *absent = 0; present = 1.*
2. ***Posterior lateral spinnerets, total length:*** *short, not extending far behind abdomen, generally only apical segment visible from dorsal view = 0; long, extending behind or curving dorsally around abdomen, both apical and medial segments generally visible from dorsal view = 1.*

**Remarks:** If a genus is scored as polymorphic, that indicates that it either includes species that fall into each state (e.g., *Scotinoecus*), or that it falls on the threshold between states (e.g., *Entypesa*).

1. ***Posterior lateral spinnerets, spigot distribution:*** *spigots present on all three segments = 0; spigots absent from basal segment = 1; spigots present only on apical segment = 2.*
2. ***Leg III, size relative to leg II:*** *subequal = 0; leg III thicker and equal or greater in length.*

**Remarks:** This is a modified version of the character previously used by several authors relating to the larger size of the posterior legs relative to the anterior legs in many burrowing spiders. In practice, we found that this was ambiguous unless discretized further.

1. ***Leg III, tibia and metatarsus, spines:*** *spines in ventral series on both segments, almost as many ventral spines as on other faces combined = 0; many more spines dorsally, on tibia ventral spines absent or bristle-like, sometimes with few strong apical spines = 1.*
2. ***Leg III, patella, thorn patch (>3 prodorsal spines):*** *absent = 0; present = 1.*
3. ***Leg III, tibia, excavation:*** *absent = 0; present = 1.*
4. ***Leg III, tibia, excavation type:*** *proximal excavation only = 0; strongly sclerotised “saddle” = 1.*

**Remarks:** The ‘saddle’ is a strongly sclerotized, concave, setae-less section of cuticle, positioned on proximal tibia III and occupying over half the length of dorsal tibia III when present. This state is only present in some halonoproctid genera (*Conothele*, *Ummidia*, *Hebestatis*). A demi-saddle is a much less pronounced concavity at the proximal end of tibia III that is not strongly sclerotized for descriptions and images of both the saddle and demi-saddle states).

1. ***Legs I and II, metatarsi, chaetotaxy type:*** *only bristle-like spines present, if strong spines are present they are positioned ventrally = 0; with one or more series of strong “digging spines” positioned laterally = 1.*
2. ***Legs I and/or II, chaetotaxy, strong, stiff spines, type:*** *relatively long, stiff lateral spines = 0; short, thorn-like lateral spines = 1.*

**Remarks:** Most taxa have state 0, state 1 is markedly different, with far more numerous, thorn-like spines, and is present only in Halonoproctidae and Stasimopidae.

1. ***Legs I and/or II, scopulae:*** *absent = 0; present = 1.*
2. ***Legs I and/or II, tarsal spines:*** *absent = 0; present = 1.*
3. ***Legs III and/or IV, scopulae:*** *absent = 0; present = 1.*
4. ***Legs III and/or IV, tarsal spines:*** *absent = 0; present = 1.*
5. ***Tarsus I, claw tufts:*** *absent = 0; present = 1.*
6. ***Tarsus I, superior tarsal claws, dentition reduction:*** *claws with normal teeth = 0; claws with small teeth or denticles only = 1; claws edentate = 2.*
7. ***Tarsus I, superior tarsal claws, teeth rows:*** *one = 0; two = 1.*
8. ***Tarsus I, superior tarsal claws, dentition type:*** *several teeth in a row = 0; one proximal tooth, sometimes with denticles = 1.*

**Remarks:** Not scored in taxa with reduced dentition (Barychelidae).

1. ***Tarsus I, inferior tarsal claw:*** *absent = 0; present = 1.*
2. ***Tarsus I, inferior tarsal claw, dentition:*** *edentate = 0; dentate = 1.*
3. ***Tarsus I, tarsal organ, shape:*** *flattened = 0; protruding, distally lobed = 1.*

**Remarks:** Not scored in taxa with reduced dentition (Barychelidae).

1. ***Legs, cuticle, pustules:*** *absent = 0; present = 1.*
2. ***Eye group, maximum width:*** *about 1/3 caput width = 0; about 1/2 caput width or wider = 1.*
3. ***Eye group, AME and ALE relative position:*** *AME advanced of ALE = 0; AME and ALE roughly in line = 1; ALE advanced of AME = 2.*
4. ***Eye group, advanced ALE, type:*** *ALEs only slightly to moderately advanced, not contiguous = 0; ALEs far advanced and contiguous = 1.*
5. ***Eye group, eye tubercle:*** *absent = 0; present = 1.*

**Remarks:** We consider an eye tubercle to be a demarcated raised section of the caput falling underneath at least two pairs of eyes (i.e., not all eyes have to sit on the tubercle).

1. ***Fovea, type:*** *closed, longitudinal = 0; pit-like or narrowly transverse and open = 1; closed, transverse = 2.*

**Remarks:** A closed longitudinal fovea is a simple longitudinal crack, and is only present in some Atypoidea, and in *Microhexura*. A closed transverse fovea is a closed crack running laterally and is present in most genera. Some taxa have a narrow, pit-like fovea that is difficult to place in either state 0 or 2, which we classify as state 1. In these taxa it is also generally difficult to assess the curvature of the fovea (e.g., recurved, straight, procurved).

1. ***Fovea, closed and transverse, curvature:*** *recurved = 0; straight to procurved = 1.*
2. ***Sternum, shape:*** *sternum edge in line with second coxa parallel = 0; sternum edge in line with second coxa narrowing anteriorly = 1.*
3. ***Sternum, posterior sigilla:*** *absent = 0; present = 1.*
4. ***Sternum, posterior sigilla, position:*** *inner-most point of sigilla closer to sternum edge than median line = 0; inner-most point of sigilla equidistant from sternum edge and median line = 1; inner-most point of sigilla closer to median line than sternum edge = 2.*
5. ***Sternum, sigilla, fused:*** *absent = 0; present = 1.*
6. ***Sternum, lateral sigilla:*** *absent = 0; present = 1.*
7. ***Maxillae, serrula:*** *absent = 0; present = 1.*
8. ***Maxillae, anterior lobe: unmodified or small = 0; anteriorly produced and sharp = 1.***

**Remarks:** We found this character very difficult to discretize but have included it due to its consistent use in previous datasets. We scored several taxa not traditionally considered to possess a lobe as state 1 or ambiguous if their lobe seemed comparable in size and shape to those seen in Theraphosidae – a family consistently scored as possessing produced lobes in previous datasets.

1. ***Maxillae, cuspules:*** *absent = 0; present = 1.*
2. ***Maxillae, cuspules, posterior extent:*** *not extending onto posterior heel = 0; extending onto posterior heel = 1.*
3. ***Maxillae, cuspules, lateral extent:*** *confined to inner quarter of maxilla length = 0; extending laterally beyond this = 1.*
4. ***Labium, shape:*** *short (length/width <0.6) = 0; intermediate (length/width between 0.6 and 0.9) = 1; long (length/width >0.9) = 2.*
5. ***Labium, cuspules:*** *absent = 0; present = 1.*
6. ***Labium, cuspules, number:*** *few (<10); many (≥10).*
7. ***Chelicerae, rastellum:*** *absent = 0; present = 1.*
8. ***Chelicerae, rastellum, type:*** *sessile = 0; on pronounced mound = 1.*
9. ***Chelicerae, retrolateral row of teeth sub-equal in size to teeth in prolateral row:*** *absent = 0; present = 1.*
10. ***Chelicerae, fang, keels:*** *absent = 0; present = 1.*

**Remarks:** Keels are two longitudinal ridges that run longitudinally down the outer surface of the fangs.

1. ***Abdomen, tergite(s):*** *absent = 0; present = 1.*

**APPENDIX S3: ANCESTRAL STATE RECONSTRUCTIONS – BRANCH-LENGTH SET AND MODEL COMPARISON**

**Table 3. Comparison of AICc scores for different branch-length sets and model combinations when performing ancestral state reconstruction of retreat construction method.**

| **Branch-length set** | **State transition model** | **AICc** |
| --- | --- | --- |
| Chronogram | Equal rates (ER) | 106.94 |
| Chronogram | Symmetrical rates (SYM) | 109.74 |
| Chronogram | All rates different (ARD) | 122.93 |
| Phylogram | Equal rates (ER) | 106.47 |
| Phylogram | Symmetrical rates (SYM) | 109.41 |
| Phylogram | All rates different (ARD) | 119.99 |

**Table 4. Comparison of AICc scores for different branch-length sets and model combinations when performing ancestral state reconstruction of retreat-entrance type.**

| **Branch-length set** | **State transition model** | **AICc** |
| --- | --- | --- |
| Chronogram | Equal rates (ER) | 145.35 |
| Chronogram | Symmetrical rates (SYM) | 163.21 |
| Chronogram | All rates different (ARD) | 211.69 |
| Phylogram | Equal rates (ER) | 143.78 |
| Phylogram | Symmetrical rates (SYM) | 162.86 |
| Phylogram | All rates different (ARD) | 207.37 |
